# Potential Barriers amongst Health Care Professionals of Pakistan in managing COVID-19 patients

**DOI:** 10.12669/pjms.36.COVID19-S4.2753

**Published:** 2020-05

**Authors:** Muhammad Irfan Ul Haq, Faraz Shafiq, Haris Sheikh

**Affiliations:** 1 Muhammad Irfan Ul Haq, MBBS, FCPS. Department of Anaesthesiology, The Aga Khan University, Stadium Road, Karachi, Pakistan; 2 Faraz Shafiq, MBBS, MCPS, FCPS. Department of Anaesthesiology, The Aga Khan University, Stadium Road, Karachi, Pakistan; 3 Haris Sheikh, MBBS. Department of Anaesthesiology, The Aga Khan University, Stadium Road, Karachi, Pakistan

**Keywords:** Pakistan, COVID 19, Health Personnel, Attitude

## Abstract

**Objectives:**

To evaluate basic knowledge of Health Care Professionals (HCPs) of Pakistan in managing COVID 19 patients. It includes information regarding infection control measures, administrative and professional support. This was followed by evaluation of psychological factor that can act as a barrier in effective management of these patients.

**Methods:**

The survey was conducted on line using Google Form. After approval from hospital ethical committee survey link was disseminated to HCPs using social media.

**Results:**

Four hundred fifteen HCPs were participated. Most of them were younger than 30 years and majority of them were postgraduate trainees. Results showed gaps in the knowledge about basic infection control measure like donning/doffing and understanding about high-risk procedures. On job training, professional and administrative support is compromising. Many of HCPs are anxious nowadays, having symptoms related to burn out with logical reasons behind. Even with all those hurdles they are committed and ready to volunteer themselves.

**Conclusion:**

The HCPs of Pakistan needs urgent attention for providing them Formal training regarding infection control measure. Administrative and professional support is required from institutions and scientific societies. Online teaching modules and webinar is a suitable option. The symptoms of burn out are significant and would increase with passage of time. This needs to be supported by occupational health committees.

## INTRODUCTION

The 2019 coronavirus disease (COVID 19) is now become a global health threat. The World Health Organization (WHO) already declared the outbreak, a public health emergency of international concern.^1^ It started from China in December 2019, and affected almost 210 countries. According to WHO, total confirmed cases are 2,475,743, and a death toll of 169,151 deaths over the time. With 20,130 confirmed cases and 459 total deaths till May 3, 2020, the number of cases is growing significantly in Pakistan. (Daily DAWN May 4, 2020) We, the current generation of Health Care Providers (HCPs) have never seen this type of pandemic before, hence not well prepared. Data from developed countries has shown psychological impact on HCPs leading to feeling of depression, fear and insomnia.^2^ To the greater extent symptoms were directly related to risk of getting infection and inadequate protection from contamination.^3^ Low middle-income countries (LMICs) like Pakistan, cannot afford strict contagion measures due to economical reasons. Moreover, our social setup, religious beliefs and educational background, is much different from advanced world. So, we should be expecting increased exposure of HCPs, both to symptomatic and asymptomatic careers. With limited health care facilities and available expertise, the gravity of this would be very severe in Pakistan. We more often discuss about the availability of Personal Protective Equipment (PPEs). Though these are mandatory requirements, but effective management needs implementation of processes and planning.^4^ Individual teams should have adequate training, administrative and professional support, not only to deal with this crisis but also to keep them healthy. This survey was designed to assess the problems; which HCPs are facing during this pandemic. The primary objective was to evaluate the basic knowledge of HCPs regarding infection control measures. This was followed by the evaluation teaching and training in context to infection control measures and provision of administrative and professional support. The secondary objective was to see the psychological impact of this on HCPs, which can act as a barrier in effective management of these patients.

## METHODS

Exemption from Ethical review committee of (Ref# 2020-4744-10172, Dated: 17 April, 2020) Aga Khan University was taken for conducting this survey. The survey was designed using “**ON line - *GOOGLE FORM”*.** The survey consists of a set of questions focusing on:

***1: Demographics:*** Age, sex, marital status, association with any comorbid condition.
***2. Occupational Background:*** Health care job status, specialty, administrative setup, years of experience.
***3. Region:*** City and Province.
***4: Administrative Support:*** Availability of infection control department, PPEs, training regarding donning and doffing, availability of hand washing, sanitizers and shower facilities.
***5: Knowledge of HCPs about infection control measures:*** Awareness regarding donning and doffing, aerosol generating high-risk procedures and effective utilization of PPEs were questioned.
***6: Ongoing professional updating:*** Role of social media, Internet, web resources and professional societies for keeping HCPs updated.
***7: Anxiety and related factors:*** Questions related to anxiety, feeling of burn out, and performance at work.

Survey Form was distributed using GOOGLE LINK. This was done by investigators using social media, which includes Whatsapp, Twitter, and Facebook accounts. Individual contacts were also requested to disseminate the link so that maximum HCPs can get involved. The statement regarding informed consent was disclosed. One has to be agree upon it before filling up of questionnaire. The responses were collected anonymously and individual’s identity was not disclosed.

### Statistical Analysis

Responses of participants were exported from excel to statistical packages for social science version 19 (SPSS Inc., Chicago, IL) for analysis. All of these responses were compiled in tables and bar chart. Most of the questions were closed ended therefore frequency and percentages were reported. Due to large information supplementary tables and figure were also provided separately.

## RESULTS

Total 415 HCPs participated in the survey. The demographic characteristic of participants is shown in Table-I. Majority of responders (50%) belonged to age group less than 30 years. Participation was almost equivalent for both genders. Most of them were married (60%) and had no association (71%) with any comorbid condition.

**Table-I:** Demographic characteristics of HCPs.

Variables	Frequency (%)
***Age Groups***	
<30 Years	207(50.6%)
30-50 Years	172(42.1%)
>50 Years	30(7.3%)
***Gender***	
Male	211(51.6%)
Female	198(48.4%)
***Marital Status***	
Married	253(61.9%)
Single	156(38.1%)
No known comorbid	295 (71%)
Association with any medical condition	84(20.5%)
HTN	23(5.6%)
Diabetes	17(4.2%)
Asthma	17(4.2%)
Depression	12(2.9%)
IHD	6(1.5%)
Others	32(7.8%)

The postgraduate medical students filled 31% of Forms, followed by consultants (24%) and than nurses (17%). The affiliation of respondents was both from private and government sector (52% vs 40%). The clinical experience of responders varied. Most of them (55%) had experience of less than five years; while 9.6% had an experience of more than 20 years. 34% had experience ranges from 5 to 20 years. (Table-II).

**Table-II:** Occupational status of HCPs.

	Frequency (%)
***Health Care Job Status***	
Consultant	97(24%)
Medical Officer	60(15%)
Nurse	71(18%)
Postgraduate Medical Trainee	124(31.3%)
Anaesthesia technician	23(5.9%)
Others	3 (0.8%)
***Specialty***	
Anaesthesiology	146(35.7%)
Medicine and allied	61(14.9%)
Obstetrics and Gynecology	12(2.9%)
Pediatric	22(5.4%)
Surgery and allied	55(13.4%)
Intensives	4(1%)
Radiology	4(1%)
Others	93(22.7%)
***Experience***	
<5 Years	228(55.7%)
5-10 Years	83(20.3%)
11-20 Years	59(14.4%)
>20 Years	39(9.5%)
***Health Care Setup***	
Government	159(38.9%)
Semi Government	23(5.6%)
Private	211(51.6%)
***Province***	
Sindh	296(72.4%)
Punjab	56(13.7%)
KPK	41(10%)
Baluchistan	3(0.7%)
ICT	9(2.2%)
GilgitBalatistan	4(1%)

The basic knowledge of HCPs regarding infection control measures was adequate. 96% were aware about the need of having N-95 mask to protect themselves from aerosols, 80% had information that this mask needs to be fit tested before use. The knowledge about individual protective gears is also acceptable, as more than 80% of HCPs were aware about the need of having eye shield, gown, gloves and shoe covers for managing COVID cases. However, knowledge about high-risk clinical procedures, which can cause cross contamination amongst staff, was marginal. Almost 30% of participant did not label procedures like intubation, surgical intervention, dental and eye examination as a high risk.

**Fig.1:**
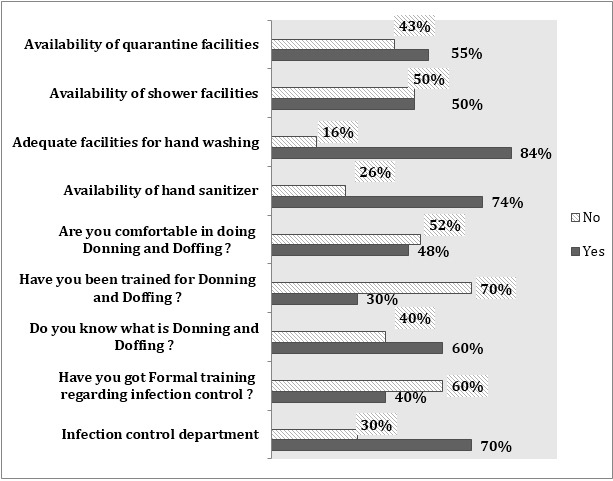
Basic Knowledge of HCPs about Infection control measures.

The department of infection control is there in almost 70% of cases. Majority of them had adequate facility hand sanitizing (99%) and washing (84%). However, only half of them (50%) have facility of shower. Facilities of quarantine were available only to 55% of cases. Full PPEs were available to only 40% of survey population. 70% had no training regarding effective use of PPEs. This is reflected by the fact that, 53% of health care staff had no formal teaching session, and about half of them (52%) were not comfortable about their technique of using protective gears. Even 41% had no idea about what is meant by donning and doffing? (Fig.1).

**Fig.2:**
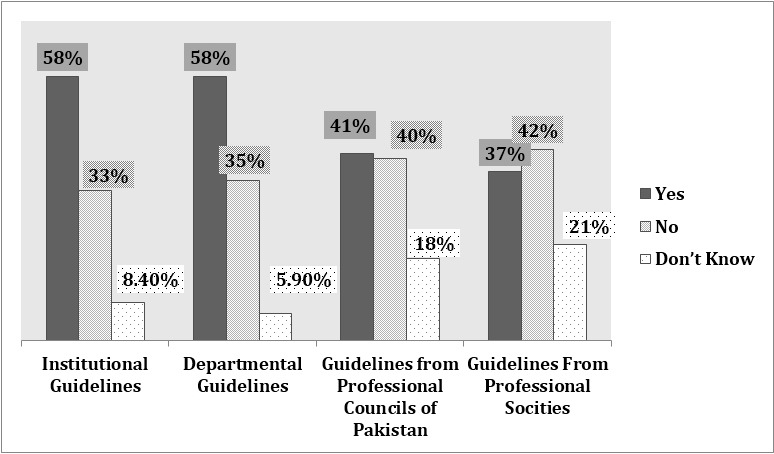
Availability of professional support to HCPs.

Professional support in the Form of management guidelines was available at departmental/institutional level in 60% of case. Similarly; majority of them (60%) were not aware about any guidelines at national level. (Fig.2). To keep them updated majority (70%) of HCPs were using online resources, followed by Face book, Google and TV media (50%). 26% had provision of lectures, likewise only 19% were using PubMed search engine.

**Fig.3:**
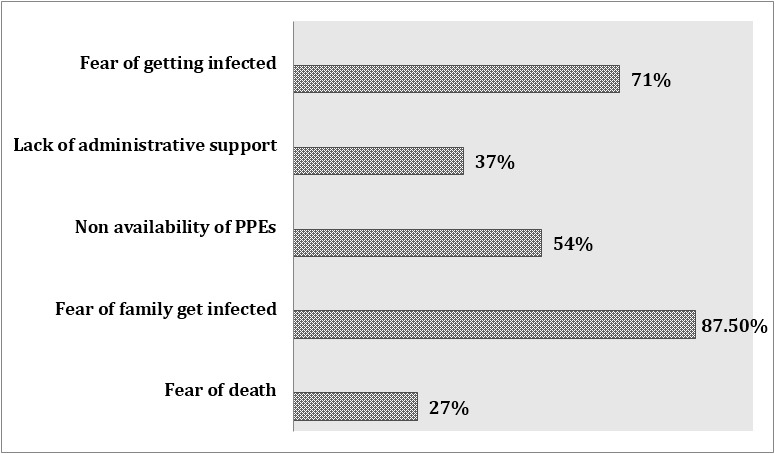
Potential barriers amongst HCPs while managing COVID-19.

**Fig.4:**
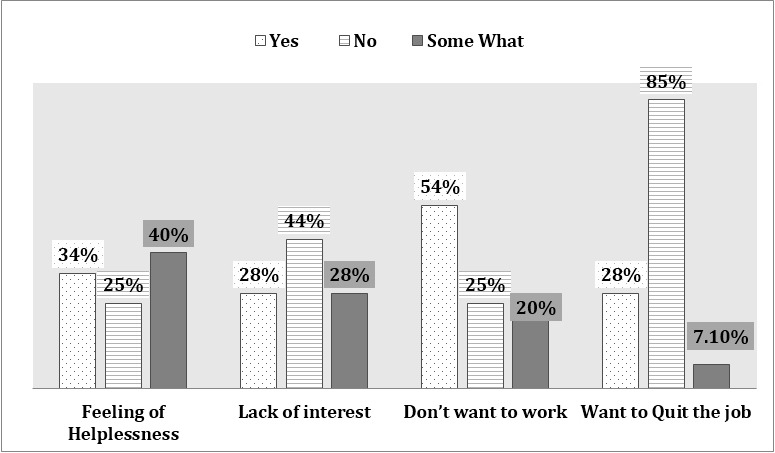
Symptoms related to burn out amongst HCPs.

With current administrative and professional support, a significant number of HCPs (66%) were not comfortable in managing COVID-19 patients. The fear of families can get infected (86%), was the major barrier against clinical care. Other factors are also mentioned in Fig.3. The symptoms related to burn out were also common amongst our HCPs. This includes feeling of helplessness, lack of interest, and don’t want to come to work or even resigning from job. (Fig.4). Occupational health facilities to employees in need were there for only 42%. With all those concerns still most of our HCPs (74%) were willing to share their expertise and volunteer themselves to other specialty.

## DISCUSSION

Based on current administrative and professional support in Pakistan, protecting HCPs from occupational exposure (COVID-19) would be very difficult. The problem is severe and we should expect large number of our HCPs getting infected. According to Daily DAWN May 4, 2020, four hundred eighty healthcare professionals in Pakistan have been exposed and diagnosed to have COVID positive. Nowadays, the whole focus of discussion is about getting PPEs. However, no one is talking about administrative support and basic prerequisites required for effective management against this pandemic. The results of our audit showed non-availability of shower facilities at many places. Similarly, the quarantine facilities were not declared in most of health care setups. Hospitals are the red zones for this infection, preventing staff from getting infected needs pragmatic approach.^5^ There were severe gap in formal training required for basic infection control measures like donning and doffing. Nonetheless, many of us are really inexperienced and are using these gears for the very first time. The answer is basic training related to infection control measures. This should include courses related to donning and doffing and monitoring those practices on priority basis.^6^ Nationwide, professional councils and societies should work together in reinforcing practice guidelines. This would be really helpful in standardizing management protocols, hence improving the outcomes.^7^ Internet facilities are widely available in Pakistan, and as depicted by our results many workers are using online resources to keep them updated. So, the best strategy would be incorporation of online teaching and training.^8^ The factors acting as a barrier; like fear of death and risk of transmitting disease to family members needs urgent attention. It’s not easy for anyone to work under these kinds of stresses and still have to manage difficult patient related scenarios. HCPs are at risk of exhaustion and burn out. The institution should device the mechanism of occupational support and professional well-being. This requires smart planning supporting trained staff, implementing infection control policies and stream lining care pathways.^9^

### Limitations of the study

The dissemination of survey Form was done unofficially using personal contacts by the investigators. This was done using social media only. The results were presented in general, and we did not evaluate the knowledge of any specific group or any relationship between the variables. The total number of 415 is not very impressive, which may reflects the culture where people don’t want to participate in quality initiatives with logical reasons behind.

## CONCLUSION

The HCPs needs urgent attention in terms of Formal training regarding infection control measures. Institutions should provide them with administrative facilities, logistics and occupational support. The Professional council and societies should support HCPs in providing practice guidelines and management protocol so that care providers can work efficiently and effectively.^10^

### Authors’ Contribution

**MIUH:** Conceived, Designed and Manuscript writing.

**FS:** ERC approval, Methodology, Data Collection, Developing the PerForma using Google Form, Manuscript writing and Statistical analysis.

**HS:** Data collection.
